# The c-Jun/RHOB/AKT pathway confers resistance of *BRAF*-mutant melanoma cells to MAPK inhibitors

**DOI:** 10.18632/oncotarget.3888

**Published:** 2015-05-07

**Authors:** Audrey Delmas, Julia Cherier, Magdalena Pohorecka, Claire Medale-Giamarchi, Nicolas Meyer, Anne Casanova, Olivier Sordet, Laurence Lamant, Ariel Savina, Anne Pradines, Gilles Favre

**Affiliations:** ^1^ Inserm, UMR 1037-CRCT, Toulouse, France; ^2^ Université Paul Sabatier, Toulouse, France; ^3^ Institut Claudius Regaud, Institut Universitaire du Cancer de Toulouse-Oncopole, Laboratory of Medical Biology and Oncogenetics, Toulouse, France; ^4^ Centre Hospitalo-Universitaire de Toulouse, Institut Universitaire du Cancer de Toulouse-Oncopole, Department of Dermatology, Toulouse, France; ^5^ Centre Hospitalo-Universitaire de Toulouse, Institut Universitaire du Cancer de Toulouse-Oncopole, Department of Pathology, Toulouse, France; ^6^ Scientific Partnerships, Roche SAS, Boulogne Billancourt, France

**Keywords:** melanoma, RHOB, AKT, vemurafenib, resistance

## Abstract

The response of *BRAF*-mutant melanoma patients to BRAF inhibitors is dramatically impaired by secondary resistances and rapid relapse. So far, the molecular mechanisms driving these resistances are not completely understood. Here, we show that, in *BRAF*-mutant melanoma cells, inhibition of BRAF or its target MEK induces RHOB expression by a mechanism that depends on the transcription factor c-Jun. In those cells, RHOB deficiency causes hypersensitivity to BRAF and MEK inhibitors-induced apoptosis. Supporting these results, loss of RHOB expression in metastatic melanoma tissues is associated with an increased progression-free survival of *BRAF*-mutant patients treated with vemurafenib. Following BRAF inhibition, RHOB activates AKT whose inhibition causes hypersensitivity of *BRAF*-mutant melanoma cells to BRAF inhibitors. In mice, AKT inhibition synergizes with vemurafenib to block tumor growth of *BRAF*-mutant metastatic melanoma. Our findings reveal that BRAF inhibition activates a c-Jun/RHOB/AKT pathway that promotes tumor cell survival and further support a role of this pathway in the resistance of melanoma to vemurafenib. Our data also highlight the importance of using RHOB tumor levels as a biomarker to predict vemurafenib patient's response and to select those that would benefit of the combination with AKT inhibitors.

## INTRODUCTION

The hypothesis of melanoma addiction to the RAF/MEK/ERK pathway, namely the MAPK pathway, emerged with the discovery of a high frequency of the BRAF^V600E^-activating mutation in melanoma cell lines and primary tumors [1]. Since then, evidence of melanoma dependency on MAPK pathway accumulated and its inhibition by either BRAF or MEK inhibitors (BRAFi and MEKi, respectively) emerged as a therapeutic strategy [2]. The first-in-class specific BRAFi, vemurafenib (PLX4032), led to an unprecedented response for 80% patients in phase III clinical trials [3, 4]. However, the improvement of disease-free survival and global survival was disappointingly weak, due to the rapid acquisition of resistance, opening the way to the discovery of new classes of MAPK inhibitors [5] or to BRAFi combinations with cytotoxic drugs [6].

Intensive investigations led to identify several resistance mechanisms on the basis of *in vitro* resistant cell lines and clinical studies. Most of them reactivate the MAPK and/or PI3K/AKT pathways by acquisition of *RAS* and *MEK* mutations [7, 8], or over-expression of COT [9], CRAF or growth factor receptor such as FGFR3, EGFR, PDGFRα, PDGFRβ [7, 10–13]. Moreover, MAPK inhibition modulates transcription factors expression that could regulate adaptive responses [14]. Although their roles are not clearly defined, the genes whose expression is modulated could impact on the sensitivity of melanoma cells to BRAFi and therefore could be of clinical relevance. In line with this hypothesis, the transcription factor FOXD3 and the pro-apoptotic BH3-only protein NOXA, are induced and down-regulated, respectively following PLX4032 treatment thus promoting resistance to cell death [15, 16]. Hence, MAPK inhibition can regulate *per se* the expression of resistance factors.

RHOGTPases are regulated by many signaling pathways, such as the MAPK pathway, and control numerous cellular functions including the balance between survival and apoptosis [17]. In that way, they may impact on the cellular response to MAPK inhibitors. Cross-regulations between RHOGTPases and MAPK pathways have been reported [18, 19] and several members of the RHOGTPase family have been involved in apoptosis inhibition to both chemotherapies and targeted therapies. For instance, RHOJ mediates melanoma cell resistance to dacarbazine [20], RAC1 is involved in breast cancer cell response to trastuzumab [21] and RHOE/RND3 enhances multidrug resistance in gastric cancer cells [22]. In addition, inhibition of MAPK pathway has an impact on the regulation of the expression of RHOGTPase genes. This may result in a modulation of the tumor cell sensitivity to MAPK inhibitors, as demonstrated for RHOE/RND3, which impedes melanoma cell invasion in response to PLX4032 [23].

We therefore investigated the role of RHOGTPases in melanoma cell response to PLX4032 and others inhibitors of the MAPK pathway. Using RT-qPCR screening, we detected a significant induction of RHOB expression upon PLX4032 treatment in *BRAF*-mutant melanoma cells. RHOB is well known to be modulated in response to anticancer therapies and to control tumor cell response to ionizing radiation and cytotoxic drugs [24–26]. Moreover, RHOB has also been involved in the response to targeted therapy as described by Vishnu and colleagues in ovarian cells [27]. Here, we show that in *BRAF*-mutant melanoma cells, PLX4032 induces c-Jun activation resulting in RHOB expression and subsequent AKT activation. We demonstrate that pharmacological association of BRAFi with AKT inhibitors (AKTi) displays a higher antitumor activity than BRAFi alone on *BRAF*-mutant cells. Finally, we demonstrate that the expression of RHOB in melanoma tissues determines a poor clinical response to PLX4032. Altogether, these findings support a potential role for RHOB as a predictive biomarker to PLX4032 therapy and the c-Jun/RHOB/AKT signaling axis as a new target to prevent resistance to BRAFi of *BRAF*-mutant melanoma tumors.

## RESULTS

### RHOB expression is induced by the BRAFi PLX4032 in BRAF-mutant melanoma cells

**Figure 1:**
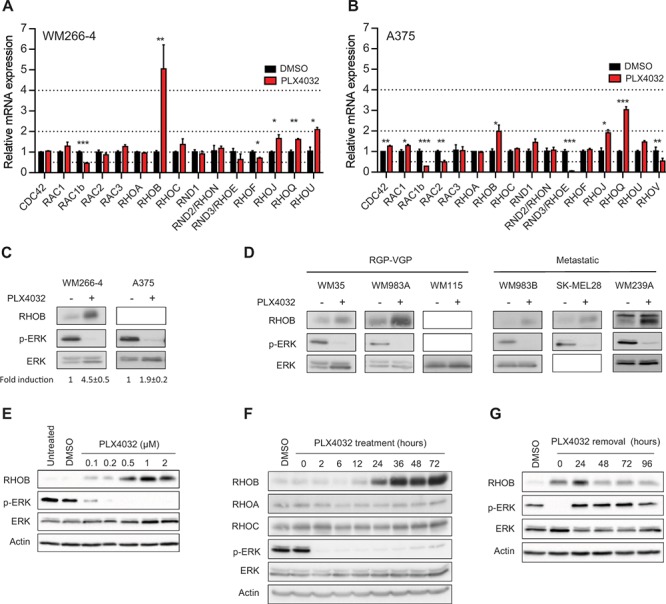
PLX4032 increases RHOB expression in BRAF^V600E^ melanoma cells **A** and **B.** RT-qPCR analysis of RHOGTPase transcripts in WM266-4 and A375 cells treated with 1 μM PLX4032 for 48 h. **C** and **D.** Western blotting of RHOB, p-ERK and ERK in *BRAF*-mutant melanoma cells treated with 1 μM PLX4032 for 48 h. **E** and **F.** Western blotting of the indicated proteins in WM266-4 cells treated with the indicated PLX4032 concentrations for 48 h (E) and for the indicated times with 1 μM PLX4032 (F). **G.** WM266-4 cells were treated with 1 μM PLX4032 for 48 h, then washed and cultured in PLX4032-free medium for the indicated times. RHOB, RHOA, RHOC, p-ERK and ERK were analyzed by Western blotting. Actin was the loading control.

We first analyzed RHOGTPase transcripts in the metastatic BRAF^V600X^-mutant melanoma cell lines WM266-4 and A375 following a 48 h treatment with PLX4032. In both cell lines, RAC1b mRNA levels decreased while those of RHOB, RHOJ and RHOQ increased (Figure 1A and 1B). *RHOB* was the most induced gene with a factor of 5.7 ± 1.2 in WM266-4 cells (Figure 1A) and of 2.0 ± 0.3 in A375 cells (Figure 1B). In those two cell lines, the increase in the RHOB mRNA level was associated with an increase in the RHOB protein level (Figure 1C). PLX4032 treatment also increased RHOB protein level in six other *BRAF-*mutant melanoma cell lines, which are representative of melanoma progression (RGP-VGP to metastatic) (Figure 1D).

The increase in RHOB expression in WM266-4 cells treated with PLX4032 for 48 h was dose-dependent, reaching a maximum at 1 μM, and was coincident with the inhibition of ERK phosphorylation (Figure 1E and 1F). At this PLX4032 concentration, RHOB induction was clearly detected after 24 h and increased over time (Figures 1F and S1). In contrast, the two RHOB homologs RHOA and RHOC were not induced (Figures 1F and S1). To determine whether continuous exposure to PLX4032 was necessary to maintain the observed elevated expression of RHOB, we examined RHOB expression following PLX4032 removal. We found that RHOB expression was maintained and did not return to its baseline levels even 96 h after termination of the PLX4032 treatment (Figure 1G). As a control, ERK phosphorylation was recovered after 24 h indicating that BRAF inhibition was indeed obliterated (Figure 1G). These results suggest that although BRAF inhibition is required to prime RHOB induction, an adaptive mechanism of melanoma cells may occur following PLX4032 treatment to maintain high levels of RHOB. We conclude that PLX4032 triggers a sustained induction of RHOB expression in *BRAF*-mutant melanoma cells.

### MAPK inhibition triggers RHOB induction in BRAF-mutant melanoma cells

**Figure 2:**
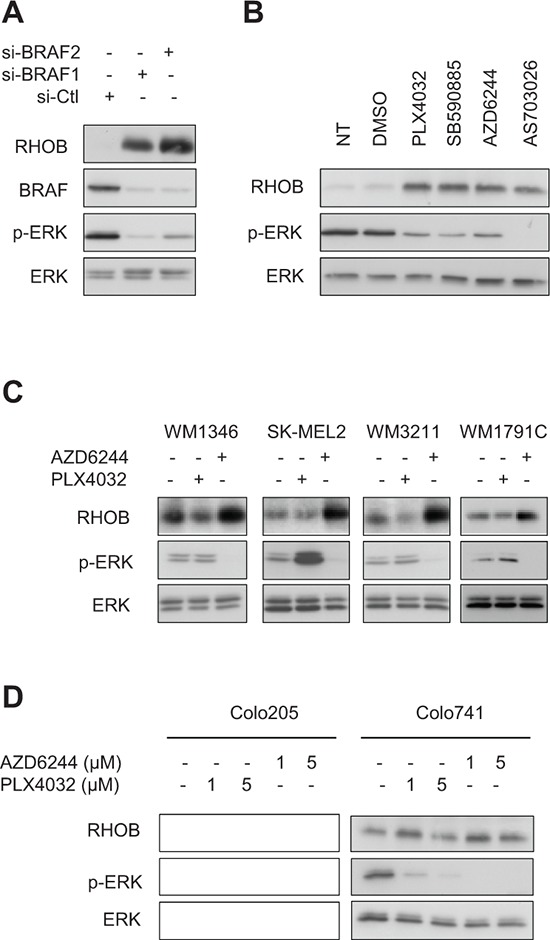
Inhibition of the BRAF/MEK/ERK pathway induces RHOB in melanoma cells **A** and **B.** Western blotting of the indicated proteins in WM266-4 cells transfected with BRAF-targeting (si-BRAF1 and si-BRAF2) or non-targeting (si-Ctl) siRNAs (A) or treated for 48 h with 1 μM of BRAF (PLX4032 or SB590885) or MEK (AZD6244 or AS703026) inhibitors (B). **C.** Western blotting of RHOB, p-ERK and ERK in wild-type *BRAF* melanoma cell lines treated with 1 μM PLX4032 or AZD6244 for 48 h. **D.** Western blotting of RHOB, p-ERK and ERK in BRAF^V600E^ colon cell lines treated with the indicated PLX4032 or AZD6244 concentrations for 48 h.

Next we addressed whether RHOB induction by PLX4032 in *BRAF*-mutant melanoma cells was related to BRAF inactivation. We found that BRAF inhibition with siRNA or with the PLX4032-unrelated BRAF^V600E^ inhibitor SB590885 increased RHOB protein levels in WM266-4 cells (Figure 2A, 2B). Inhibition of the BRAF substrate MEK with AZD6244 or AS703026 also resulted in an increased RHOB expression in those cells (Figure 2B). These results indicate that BRAF inactivation causes RHOB overexpression in *BRAF*-mutant melanoma cells.

In melanomas, the MAPK pathway is frequently hyperactivated by mutations in the *BRAF* gene (approximately 50% of melanomas) but also in *NRAS* (18%), *KIT* (9%), *HRAS* (2%) or *KRAS* (2%) genes (COSMIC database). We therefore examined the impact of PLX4032 and MEKi on RHOB expression in wild type *BRAF* melanoma cells harboring mutations in *NRAS* (WM1346 and SK-MEL2 cells), *KIT* (WM3211 cells) or *KRAS* (WM1791C cells). Consistent with the selectivity of PLX4032 for *BRAF*-mutant cells, it failed to inhibit MAPK signaling in these four cell lines, as shown by the lack of decrease in ERK phosphorylation, and the lack of RHOB induction (Figure 2C). In contrast, the MEKi AZD6244 inhibited ERK phosphorylation and induced RHOB expression (Figure 2C). Taken together, these results indicate that the BRAF/MEK pathway regulates RHOB expression.

The induction of RHOB after MAPK pathway inhibition suggests that this phenomenon could be a common feature for *BRAF*-mutant tumor cells. We therefore tested the effect of PLX4032 and AZD6244 treatment on BRAF^V600E^-mutant colon cancer cells. As shown in Figure 2D, efficient inhibition of MAPK signaling with PLX4032 or AZD6244, demonstrated by inhibition of ERK phosphorylation, did not significantly affect RHOB expression even with a higher dose (5 μM) than that used in melanoma cells (1 μM). These results suggest that RHOB modulation by MAPK signaling is not a common mechanism in *BRAF*-mutant cells but a feature of specific tumors such as melanoma.

### c-Jun is involved in PLX4032-induced RHOB expression

**Figure 3:**
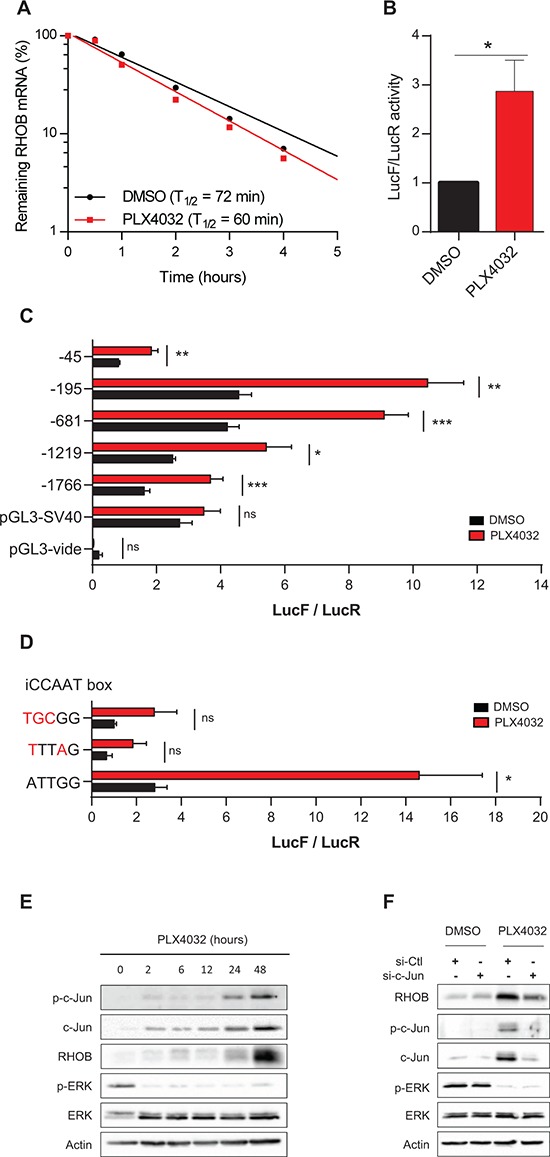
RHOB is induced by PLX4032 through transcriptional activation in an iCCAAT and c-Jun dependent pathway **A.** After a 48 h incubation of WM266-4 cells with PLX4032 (1 μM), RHOB mRNA half-life was measured by RT-qPCR using the transcription inhibitor actinomycin D (5 μg/mL) and normalized to the long half-life of actin mRNA (one representative experiment). **B-D.** Luciferase assays in WM266-4 cells co-transfected with RHOB promoter-firefly luciferase constructs and pRL-CMV before treatment with PLX4032 (2 μM, 24 h in B and C; 1 μM, 48 h in D). **E** and **F.** Western blotting of p-c-Jun, c-Jun, RHOB, p-ERK and ERK in WM266- 4 cells treated with 1 μM PLX4032 for the indicated times (E) or transfected with c-Jun-targeting or non-targeting (si-Ctl) siRNAs before treatment with 1 μM PLX4032 for 48 h F. Actin was the loading control.

We next investigated the mechanism underlying RHOB induction by PLX4032. Increase of RHOB transcript stability has been reported in response to UV [28, 29] and camptothecin [30]. Experiments performed in the presence of the transcription inhibitor actinomycin D showed that the half-life of RHOB mRNA was not significantly prolonged in PLX4032-treated cells (Figure 3A). We then analyzed RHOB promoter activity using a luciferase reporter gene assay system. We found a 2 to 3-fold increase of RHOB promoter activity after a 24 h PLX4032 treatment (Figure 3B).

To identify the promoter region involved in the transcriptional activation of RHOB in response to PLX4032, we performed luciferase assays with plasmids containing different RHOB promoter lengths. We found that all constructs increased luciferase activity following PLX4032 treatment, including the −45 shorter one, which contains only the TATA boxes and an inverted CCAAT box (iCCAAT) (Figure 3C). Mutations of iCCAAT strongly reduced promoter response to PLX4032 (Figure 3D). These results indicate that this promoter region is required for RHOB induction by PLX4032.

The iCCAAT region has been reported to control RHOB expression in cell stress response in a c-Jun-dependent manner [31–33]. Hence, we examined c-Jun activation after PLX4032 treatment by analyzing both total and phosphorylated (S63) c-Jun. Figure 3E shows that both c-Jun and p-c-Jun (S63) were induced in response to PLX4032. As for RHOB, total and phosphorylated c-Jun were maintained over at least 96 h after PLX4032 removal (Figure S2A). To assess more directly the role of c-Jun in RHOB upregulation, we inhibited its expression. We found that siRNA-mediated depletion of c-Jun prevented the induction of RHOB in response to PLX4032 (Figure 3F).

Previous work show that JNK and p38 are implicated in the activation of the c-Jun/RHOB axis in response to UV and γ-radiation [32, 34]. However, we found that phosphorylated p38 and JNK rather decreased following PLX4032 treatment (Figure S2B). In addition, the JNK inhibitor, SP600125, as well as the p38 inhibitors, SB203580 and BIRB796, did not prevent PLX4032-induced c-Jun and RHOB expression (Figure S2C and S2D). Together, these data indicate that PLX4032 induces c-Jun expression and phosphorylation by a JNK- and p38- independent mechanism.

### Inhibition of RHOB sensitizes melanoma cells to MAPK inhibitors-induced apoptosis

**Figure 4:**
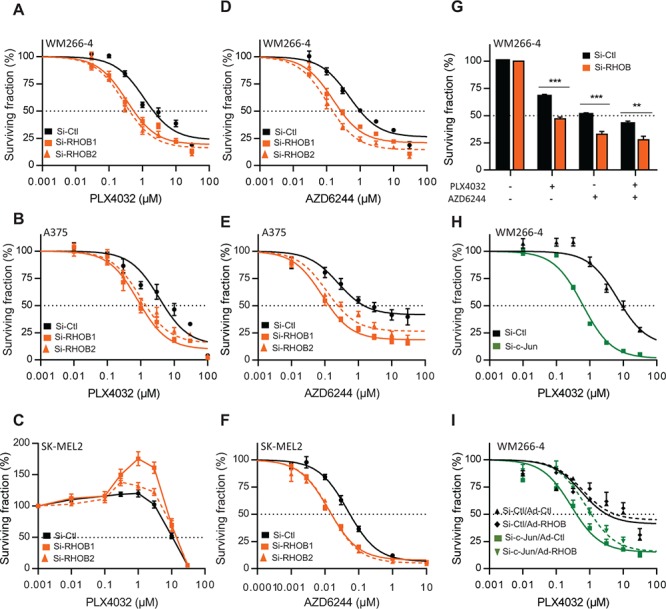
Inhibition of the c-Jun/RHOB axis increases cell sensitivity to BRAF and MEK inhibitors **A-F.** WM266-4, A375 or SK-MEL2 cells were transfected with siRNAs control (si-Ctl) or targeting RHOB (si-RHOB1 and siRHOB2) before treatment with PLX4032 or AZD6244 for 72 h. **G.** WM266-4 cells were transfected with siRNAs control (si-Ctl) or targeting RHOB (si-RHOB) before treatment with PLX4032 (1 μM) and/or AZD6244 (1 μM) for 72 h. **H.** WM266–4 cells were transfected with siRNAs control (si-Ctl) or targeting c-Jun (si-c-Jun) before treatment with PLX4032 for 72 h. **I.** WM266–4 cells were transfected with siRNAs control (si-Ctl) or targeting c-Jun (si-c-Jun), then transduced with adenovirus control (Ad-Ctl) or expressing RHOB (Ad-RHOB) and treated for 72 h with PLX4032. In each condition, cell viability was measured by MTS and the dose-response was analyzed (except in **G**).

To determine the role of RHOB in the cellular response to MAPK inhibitors, RHOB expression was prevented by RNA interference (Figure S3A). Following RHOB depletion, the IC50 of PLX4032 was significantly reduced in the *BRAF-*mutant A375 and WM266-4 cells but not in the wild-type *BRAF* SK-MEL2 cells which are insensitive to PLX4032 (Figure 4A, 4B and 4C and Table S1). In contrast, RHOB depletion sensitized both mutant and wild-type *BRAF* cells to the MEKi AZD6244 (Figure 4D, 4E and 4F and Table S1). Likewise, RHOB downregulation also sensitized WM266-4 cells to the combination of BRAFi with MEKi (Figure 4G). Because we found that c-Jun induces RHOB (Figure 3), we examined whether c-Jun inhibition would also sensitize cells to PLX4032. We found that depletion of c-Jun with siRNA sensitized WM266-4 cells to PLX4032 (Figure 4H and Table S2) and that this effect was in part reversed by adenovirus-mediated RHOB overexpression (Figures 4I and S3B and Table S3).

**Figure 5:**
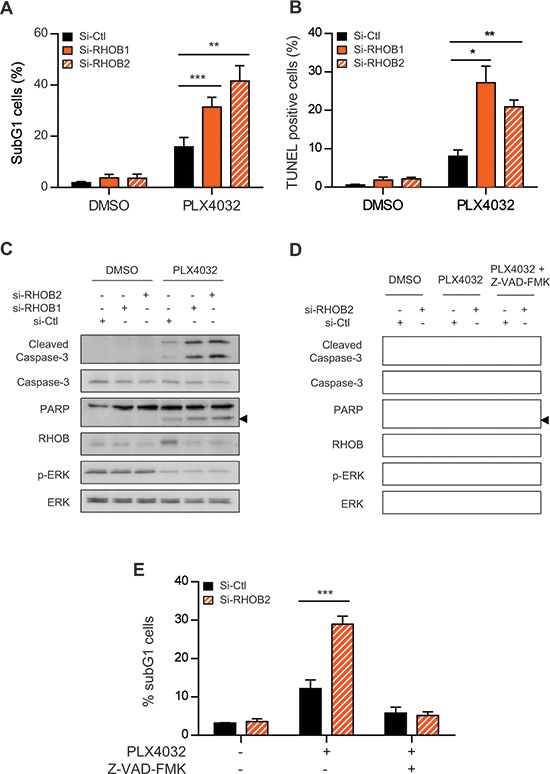
Concurrent inhibition of MAPK with RHOB triggers apoptosis WM266-4 cells were transfected with siRNAs control (si-Ctl) or targeting RHOB (si-RHOB1 and si-RHOB2) before treatment with 2 μM PLX4032 for 72 h. Apoptosis was determined by the percentage of cells with subG1 DNA content **A, E.** or positive for TUNEL assay **B**, or by Western blotting analysis of cleaved caspase-3 and cleaved PARP (the arrowheads at right indicate the cleaved fragment) **C** and **D.** Total caspase-3, RHOB, p-ERK and ERK were analyzed in parallel. In **D** and **E**, cells were co-treated or not with 2 μM PLX4032 and the pan-caspase inhibitor Z-VAD-FMK (25 μM).

To study the mechanisms underlying RHOB-dependent cell sensitivity to PLX4032, we assayed apoptotic markers after RHOB depletion. We found that RHOB siRNA increased apoptosis of WM266-4 cells in response to PLX4032 as demonstrated by an increase in the number of nuclei with subG1 DNA content, in apoptotic DNA fragmentation and in PARP and caspase-3 cleavage (Figure 5A-5C). Similar results were obtained in A375 cells (Figure S4). In addition, the pan-caspase inhibitor Z-VAD-FMK prevented PLX4032-induced PARP and caspase-3 cleavage (Figure 5D) and the accumulation of subG1 cells (Figure 5E). Overall these data show that RHOB depletion triggers caspase-dependent apoptosis of *BRAF*-mutant melanoma cells exposed to MAPK inhibitors.

### High basal RHOB expression in melanoma cell lines and patients biopsies is associated with a poor response to PLX4032

**Figure 6:**
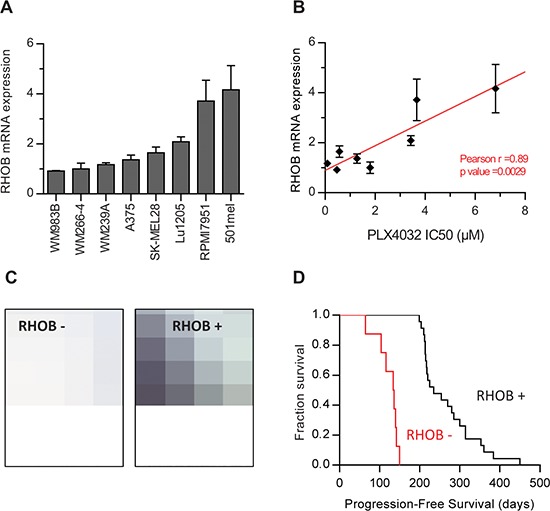
High RHOB expression in melanoma cell lines and patient melanoma biopsies predict low sensitivity to the BRAF inhibitor PLX4032 **A.** RHOB expression in a panel of *BRAF*-mutant metastatic melanoma cell lines analyzed by RT-qPCR. **B.** The IC50 of PLX4032 in a panel of *BRAF*-mutant metastatic melanoma cell lines was determined by viability assay and correlated with RHOB mRNA expression. **C.** Example of positive and negative RHOB immunostaining in melanoma biopsies. **D.** Kaplan-Meier progression-free survival of 32 patients with a metastatic BRAF^V600X^ melanoma following treatment with PLX4032 according to biopsies' RHOB staining.

Given that RHOB depletion determines the cellular response to PLX4032 in *BRAF*-mutant melanoma cells, we hypothesized that the basal expression of RHOB may predict the response to BRAFi. Therefore, we compared RHOB mRNA levels (Figure 6A) with the IC50 of PLX4032 (Figure 6B) in 8 different cell lines. A strong correlation was observed between RHOB basal expression and PLX4032 IC50. Cell lines with low RHOB expression were more sensitive to PLX4032, suggesting that, in melanoma cells, high RHOB expression may predict a poor response of *BRAF*-mutant patients to PLX4032.

RHOB expression levels were subsequently analyzed by immunostaining in a retrospective series of 32 biopsies from patients with metastatic melanoma harboring BRAF^V600X^ mutations, receiving PLX4032 as a front-line therapy (Figure 6C). We observed a significant expression of RHOB in 8 out of the 32 samples (Table S5). Interestingly, we found that the Progression Free Survival (PFS) was significantly shorter in patients whose tumor samples displayed a positive RHOB staining before treatment compared to those with negative RHOB staining (median PFS: 135 days [Interquartile range – IQR: 107; 141] versus 235 days [IQR: 214; 314] respectively; *p* < 10^−3^) (Figure 6D and Table S5), with a hazard ratio of short-duration therapeutic response of 10^−3^ (95% confidence interval – 95% CI: 2 × 10^−4^; 9 × 10^−3^). We conclude that the basal expression of RHOB in tumor cells may represent a predictive value of PLX4032 response to treatment in BRAF^V600X^ metastatic melanoma.

### RHOB modulates response to PLX4032 through AKT pathway

**Figure 7:**
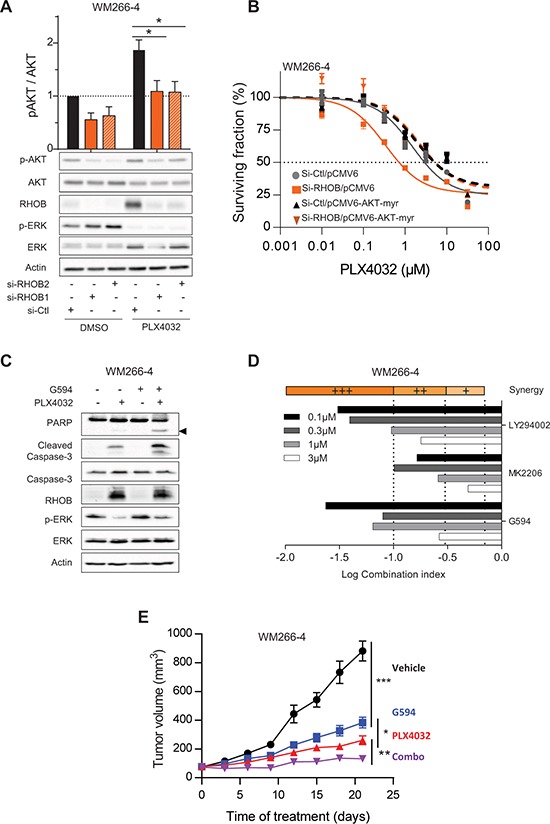
RHOB-mediated resistance to PLX4032 is overcomed by combination with an AKT inhibitor **A.** WM266-4 cells were transfected with siRNA control (si-Ctl) or targeting RHOB (si-RHOB1 or si-RHOB2) before treatment with 2 μM PLX4032 for 72 h. AKT phosphorylation was analyzed by Western blotting. Total AKT, RHOB, p-ERK and ERK were examined in parallel. Actin was the loading control. Histogram is a quantification of 5 independent experiments. **B.** WM266-4 cells were co-transfected with siRNAs control (si-Ctl) or targeting RHOB (si-RHOB2) and empty plasmid (pCMV6-Ctl) or encoding AKT-myr (pCMV6-AKT-myr). Cells were then treated with PLX4032 and the viability was assayed 72 h later. **C.** Western blotting of caspase-3 and PARP cleavage (the cleaved fragment is indicated by the arrowhead) in WM266-4 cells treated with 2 μM PLX4032 and/or 1 μM G594 for 48 h. Total caspase-3, RHOB, p-ERK and ERK were examined in parallel. Actin was the loading control. **D.** WM266-4 cells were treated with equal amount of PI3K/AKT inhibitors (LY294002, MK2206 or G594) and PLX4032. Viability was assayed 72 h later by MTS assay. Combination indexes (CI) were calculated with CompuSyn (very strong synergism (+++) CI < 0.1; strong synergism (++) 0.1 < CI < 0.3; synergism (+) 0.3 < CI < 0.7). **E.** Athymic mice were subcutaneously inoculated with WM266-4 cells. Mice were randomized 10 days later in 4 groups (10 mice per group) and treated orally with vehicle, G594 or/and PLX4032 for 21 days. The tumor volume was evaluated every 3 days.

Lastly, we investigated the molecular mechanisms by which RHOB impairs an efficient response to PLX4032. It has been previously reported that RHOB activates the AKT pathway [28, 35–37] and that AKT is activated following PLX4032 treatment [38]. We demonstrate that phosphorylated AKT is markedly reduced in response to simultaneous RHOB depletion and PLX4032 treatment in *BRAF*-mutant cells WM266- 4 (Figure 7A), Lu1205, WM239A, RPMI-7951 (Figure S5). Same findings were observed in *NRAS*-mutant cells WM1346 treated with the MEKi AZD6244 (Figure S5). These data suggest that RHOB inhibition impedes AKT signaling. Therefore the resistance to PLX4032 should be recovered in RHOB knockdown cells when AKT phosphorylation is rescued by expressing a constitutively active AKT-myr protein. Consistent with this hypothesis, overexpression of AKT-myr in RHOB depleted cells completely abolished sensitization to PLX4032 (Figures 7B and S6 and Table S4).

These findings highlight that AKT inhibition would reverse RHOB-mediated PLX4032 resistance. In order to confirm this assumption, we analyzed the combination of PLX4032 with AKT inhibitors, either G594 (an ATP competitive inhibitor of AKT provided by Genentech) or MK2206. AKT inhibition enhanced PLX4032 cytotoxic effect as shown by an increased PARP and caspase-3 cleavage (Figures 7C and S7). To determine whether combination of PI3K/AKTi and BRAFi displays additive or synergistic effect, co-treatments were performed *in vitro* and combination indexes were calculated. All three PI3K/AKT pathway inhibitors (LY294002, MK2206 and G594) demonstrated a substantial synergistic effect when combined with PLX4032 in WM266-4 cells (Figure 7D). In a same manner, a synergistic effect was found between the AKTi, G594 and PLX4032 in the 7 other *BRAF*-mutant cell lines and between G594 and AZD6244 in 2 *NRAS*-mutant cell lines (Table S6).

We next evaluated this combination effect in the WM266-4 xenografted mouse model. After tumor appearance, mice were treated with control vehicle, G594 and/or PLX4032 and the tumor growth was monitored. As shown in Figures 7E and S8, both tumor growth and tumor weight were significantly reduced in mice receiving BRAFi/AKTi combination compared to the monotherapies (Figures 7E and S8), confirming that AKT signaling pathway plays a role in the *in vivo* responses of *BRAF*-mutant tumors to PLX4032.

We conclude that PLX4032-induced RHOB expression drives AKT-dependent cell survival and that BRAFi/AKTi combination should improve therapeutic response of RHOB positive tumors.

## DISCUSSION

In this study we demonstrate that inhibition of mutant *BRAF* in melanoma cells with pharmacological inhibitors or siRNA knockdown results in the activation of the c-Jun/RHOB/AKT signaling axis, which leads to cell survival. Interestingly, a concomitant consequence of c-Jun/RHOB/AKT activation is a decrease of PLX4032 cellular responses, entailing acquired resistance.

RHO proteins have been shown to play a role in modulating cell survival and some of them have been described to be regulated by the MAPK pathway [17]. Importantly, we found that the expression of 6 members of the RHOGTPase family was significantly modified in response to PLX4032 in melanoma cells. RND3 is downregulated as previously described [23] whereas RHOB, RHOJ, RHOQ and RHOU are induced. While nothing is known about the role of RHOQ and RHOU in melanomagenesis, RHOJ participates to chemoresistance through DNA repair control [20] and modulates metastasis potential in melanoma [39]. Of interest is the PLX4032-induced RAC1b downregulation, suggesting that RAC1b and BRAF cooperate in melanomagenesis as previously described in colorectal cancer [40].

We focused our attention on the stress-inducible *RHOB* gene [28, 30, 33, 41, 42] that plays a negative role on tumor progression. Moreover, RHOB functions are mediated by tyrosine kinase receptors and their downstream effectors [25, 26, 43]. We have demonstrated previously that RHOB is downregulated by KRAS^V12^ in lung cancer cell lines increasing invasiveness via AKT1 [44]. More recently, we have revealed that *RHOB* knock-out is critical to determine tumor aggressiveness in a murine EGFR^L858R^-induced lung adenocarcinoma [45]. Herein, we show that BRAF^V600^ mutant causes a MAPK-dependent decrease of RHOB expression in melanoma cell lines. It is likely that the mechanism of RHOB downregulation is related to cell type and/or cell context. Indeed, Jiang et al. [46] found that AKT induced RHOB downregulation in non-melanoma cell lines. According to our cellular data, the patient's samples revealed an absence of RHOB expression in more than 70% of melanoma tissues bearing BRAF^V600E^ mutants. This suggests that reduced RHOB levels represent a critical event during melanomagenesis.

The observation of a persistent RHOB expression after PLX4032 removal, despite ERK re-phosphorylation (Figure 1G), suggests that RHOB participates to an original long lasting adaptive cellular program in melanoma cells. We show that RHOB expression after PLX4032 treatment depends on the transcription factor c-Jun. It has been demonstrated that iCCAAT is critical for UVB-induced RHOB expression [32, 47]. In that model, phosphorylated c-Jun is associated to ATF2 and NF-Y inducing HDAC1 release then allowing p300 binding to iCCAAT triggering RHOB expression. In stress conditions, c-Jun was described to be phosphorylated following JNK and p38 activation [32, 34] while in our model JNK and p38 were not involved in PLX4032-induced c-Jun phosphorylation and expression. We evidence here a key role of c-Jun in the long lasting regulation of RHOB expression by PLX4032 and the significance of this pathway in cell survival following cellular treatment.

RHOB has been demonstrated to control AKT phosphorylation to exert its cellular functions in many cell types. Here, we demonstrated that, by contrast to lung [44] and breast cancer [36] cells, RHOB downregulation decreases AKT phosphorylation in melanoma, similarly to endothelial cells [36, 48] (Figures 7A and S5). These data reinforce the notion that the modulation of AKT phosphorylation by RHOB depends on the cellular phenotype. We show here that RHOB/AKT signaling inhibition is critical for PLX4032-induced caspase-dependent apoptosis. In the same way, genotoxic stress-induced RHOB expression favors cell survival through AKT pathway [28, 30, 35, 41, 49]. We provide new evidence that the RHOB/AKT axis is a common adaptive mechanism to cell injury.

It has been previously reported that AKT is activated in PLX4032-treated PTEN negative melanoma and is involved in melanoma resistance [50]. However, the role of the c-Jun/RHOB/AKT axis in the cellular sensitivity to MAPK inhibitors appeared to be independent on PTEN status. Indeed, whereas WM266-4 cells lack PTEN expression, A375 cells are wild-type for PTEN and are also sensitized to MAPK inhibitors by RHOB siRNA (Figures 4, 5 and S4) or AKTi (Figure 7, Table S6).

The combination of PI3K/AKT inhibitors with PLX4032 to improve melanoma treatment has been recently proposed although the mechanism was not clearly established. We demonstrate that PLX4032 induces a cell adaptive response including a long lasting expression of c-Jun and RHOB leading to the activation of AKT signaling axis which may trigger cell resistance by inhibiting apoptosis. While AKTi are not able to induce cell death by themselves they lead to a synergistic synthetic lethal interaction with BRAFi. Our observations further support the rational to treat patients with a combination of BRAF and AKT inhibitors.

Finally, our results on metastatic melanoma biopsies highlight a pivotal role for RHOB in the therapeutic management of this disease. Interestingly, the analysis of RHOB expression in these samples revealed that patients with RHOB positive biopsies displayed a poor response to PLX4032. We therefore hypothesize that patients with a high RHOB expression would display a high AKT pathway activity and benefit from BRAF and AKT inhibitors combination rather than BRAFi monotherapy. However, it could not be excluded that RHOB negative patients' survival would also be improved by such a therapeutic combination by preventing potential late resistance appearance. Analyzing future combinational clinical trial regarding RHOB status will bring answers to these remaining questions.

Taken together, our findings tempt us to conclude that the c-Jun/RHOB/AKT signaling axis is a synthetic lethal partner of *BRAF*-mutant, and potentially of *NRAS*-mutant, melanomas and to propose RHOB as a critical biomarker to predict patient's response to PLX4032 and to select those that would benefit of the combination with AKT inhibitors.

## MATERIALS AND METHODS

### Cell culture and pharmacological inhibitors

WM115, WM266-4, A375, SK-MEL28, SK-MEL2 and RMPI-7951 were purchased at ATCC; WM35, WM983A, WM983B, WM239A, WM3211 and Lu1205 at Coriell Institute and WM1346 at Wistar Institute. WM115, WM266-4, A375 and SK-MEL28 were cultured in DMEM/FBS 10% (v/v), SK-MEL2, 501mel and RPMI-7951 in RPMI-1640/FBS 10% (v/v), WM35, WM983A, WM983B, WM3211, WM1346 and Lu1205 in MCDB153 medium with 20% Leibovitz L-15 medium (v/v), 2% FBS heat inactivated (v/v), 5 μg/mL insulin and 1.68 mM CaCl2. Cell lines were authenticated for mutations in *BRAF* and *NRAS* by sequencing within the time frame of the experiments. PLX4032, SB590885, AZD6244, AS703026 and MK2206 were from Selleck Chemicals, Z-VAD-FMK and actinomycin D from Sigma-Aldrich, LY294002 from Calbiochem, G594 competitive inhibitor of AKT was provided by Genentech.

### Transfection

RNA interference was achieved by transfecting siRNA with Oligofectamine^TM^ in OptiMEM^TM^ (Invitrogen) according to the manufacturer's instructions. After 6 h, transfection medium was replaced and inhibitors were added. siRNA sequences are reported in Table S7. pCMV6-AKT-myr plasmid, gifted by Pr. Franke (Columbia University) and pCMV6-CTRL were co-transfected with siRNA using Lipofectamine® 2000 in OptiMEM^TM^.

### Adenovirus transduction

Cells were first transfected with siRNA and then transduced overnight at M.O.I 25 with replication-defective (ΔE1, E3) adenoviral vectors expressing RHOB (Ad-RHOB) or control under the transcriptional control of the CMV promoter as previously described [51].

### Cell proliferation assay

After transfection or transduction, cells were treated with inhibitors. 72 h later, the relative number of viable cells was measured by incubating cells with MTS reagent (CellTiter 96® AQueous One Solution Cell Proliferation Assay from Promega) as recommended by the manufacturer. Relative cell survival in the presence of inhibitors was normalized to the untreated cells after background corrections.

### Western blot analysis

Cell extracts were analyzed by Western blotting with the following primary antibodies against RHOB, RHOA, BRAF, ERK1/2 (Santa Cruz Biotechnology); RHOC, p-ERK (T202/Y204), p-AKT (S473), AKT, PARP, Cleaved Caspase-3, Caspase-3, c-Jun, p-c-Jun (S63), p-p38 (T180/Y182), p38, pJNK (T183/Y185), JNK (Cell Signaling Technology) or actin (Chemicon). Detection was performed using peroxydase-conjugated secondary antibodies and chemi-luminescence detection kit (Clarity^TM^ ECL, Biorad) on autoradiographs or with ChemiDoc^TM^ MP Imaging System (Bio-Rad). Quantifications were done for three independent experiments with ImageLab software (Bio-Rad) and normalized to actin.

### Quantitative real-time reverse transcription–PCR

Total RNA was isolated by RNeasy® kit (Qiagen) and reverse-transcripted using iScript^TM^ cDNA synthesis kit (Bio-Rad). Quantitative PCR was performed with a CFX96^TM^ detection system (Bio-Rad) using iQ^TM^ SYBR® Green Supermix (Bio-Rad) and normalized to actin or 28S. Primers sequences are detailed in Table S8.

### Study of RHOB promoter activity

The previously described pGL3-RHOB plasmids containing different lengths of RHOB promoter sequence upstream of the firefly luciferase gene into pGL3 luciferase reporter vector [52] were co-transfected with pRL-CMV vector (Promega) as an internal control with Lipofectamine^TM^ 2000 (Invitrogen). The iCCAAT of the shorter construct (−45) was mutated using QuickChange® Site-Directed Mutagenesis Kit (Stratagene). The obtained constructs were sequenced to ensure successful mutagenesis. Firefly and renilla luciferase activities were measured with Dual-Luciferase Reporter Assay (Promega).

### Apoptosis analysis

DNA fragmentation was detected by a modified TUNEL procedure using the ApopTag® Fluorescein *In Situ* Apoptosis Detection Kit (Millipore). Positive cells were counted by fluorescence microscopy. For subG1 analysis, DNA content was assessed by staining ethanol-fixed cells with propidium iodide and monitoring by FACSCalibur^TM^ (BD Biosciences). The number of cells with subG1 DNA content was determined with the ModFit software.

### Animal studies

1.2 × 10^6^ WM266-4 cells were subcutaneously injected into the flank of 6 weeks female athymic mice (NMRI-NU, Janvier) and allowed to grow 10 days. Subsequently, animals received by oral gavage 50 mg/kg/bid of PLX4032 and/or 20 mg/kg/day G594 or vehicle. PLX4032, formulated as MBP, and G594 were provided by Genentech and prepared according to their advices. Tumor sizes were evaluated every 3 days by caliper measurement of two perpendicular diameters (l < L) and the tumor volumes were calculated with the following equation tumor volume = l × l × L/2. Animals were euthanized after 21 days of treatment, tumor were harvested and weighted. All animal procedures were performed in accordance with the animal care guidelines of the European Union and French laws and were approved by the local Ethical Commission of the Institut Claudius Regaud.

### Melanoma patient tumor samples and immunostaining

Tumor samples from 32 patients with metastatic BRAF^V600E^ melanoma (confirmed by genotyping) were obtained from the tumor bank of the department of pathology (Toulouse-Purpan Hospital, France) after written informed consent. Tumor samples consisted of 19 distant metastasis, 5 lymph nodes and 8 primary melanomas. Progression-free survival was retrospectively evaluated for each patient. Paraffin 4 μm-thick sections of melanoma biopsies were incubated with RHOB antibody (Santa Cruz Biotechnology) and revealed using the DAKO-LSAB+ peroxidase kit and diamino-benzidine (DakoCytomation).

### Statistical analysis

All results are presented as the mean  ±  standard error of the mean (SEM) for at least three independent experiments. Statistical analyses of continuous variable were done using *t*-test and IC50 calculation and comparison were performed using GraphPad Prism, version 5.01 (GraphPad Software Inc., San Diego, CA, USA). *P < 0.05; **P < 0.01; ***P < 0.001. Patient survival analysis were done using log-rank test of Kaplan-Meier survival curves. Hypotheses were two-sided, with an Alpha risk of 5%.

## SUPPLEMENTARY FIGURES AND TABLES


