# Supplementary material for: The c-Jun/RHOB/AKT pathway confers resistance of BRAF-mutant melanoma cells to MAPK inhibitors
Source: Oncotarget. 2015 May 7;6(17):15250–64. doi: 10.18632/oncotarget.3888 (PMC4558149; doi:10.18632/oncotarget.3888)
Supplement: Supplementary file 1 [file oncotarget-06-15250-s001.pdf]

SUPPLEMENTARY FIGURES AND TABLES

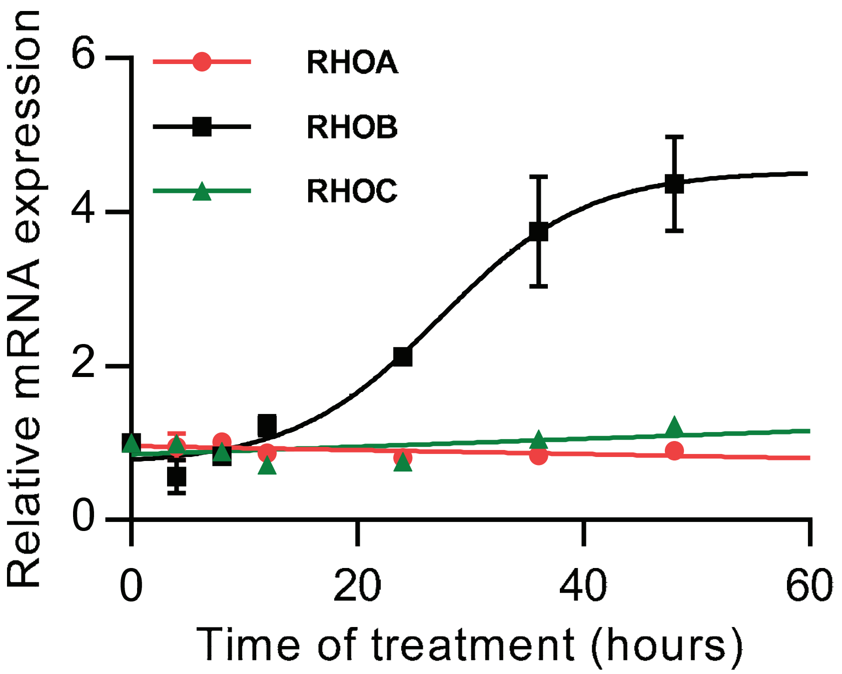

Supplementary Figure S1: PLX4032 increases RHOB mRNA levels. RHOA, RHOB and RHOC mRNA levels were assayed by RT-qPCR in response to 1 µM PLX4032 for the indicated times in WM266-4 cells.

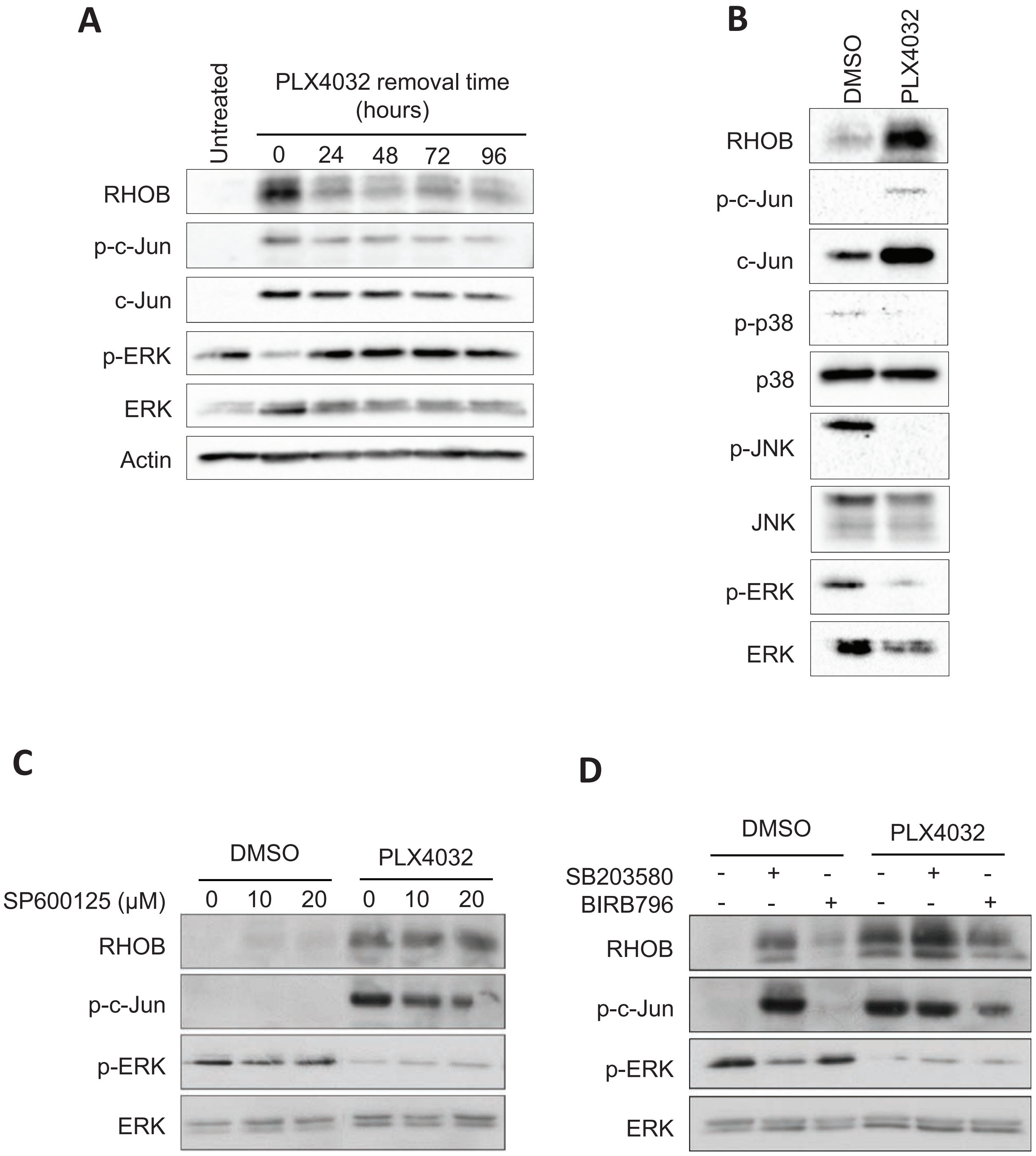

Supplementary Figure S2: PLX4032 induces long lasting activation and expression of c-Jun independently of JNK and p38. A. WM266-4 cells were treated with 1 μM PLX4032 for 48 h, then washed and cultured in PLX4032-free medium for the indicated times. RHOB, c-Jun and p-c-Jun S63 were analyzed by Western blotting, actin was the loading control. **B.** Western blotting analysis of p38, p-p38 T180/Y182, JNK, c-Jun, p-c-Jun S63, ERK and p-ERK T202/Y204 in WM266-4 cells treated with 1 μM PLX4032 for 48 h. **C.** Western blotting analysis of c-Jun, p-c-Jun S63 in WM266-4 cells treated for 48 h with the JNK inhibitor, SP600125, either alone or in combination with PLX4032 (1 μM). **D.** Western blotting analysis of RHOB, p-c-Jun S63, ERK and p-ERK T202/Y204 in WM266-4 cells co-treated for 48 h with the p38 inhibitor, SB203580 (20 μM) or BIRB796 (40 μM), and PLX4032 (1 μM).

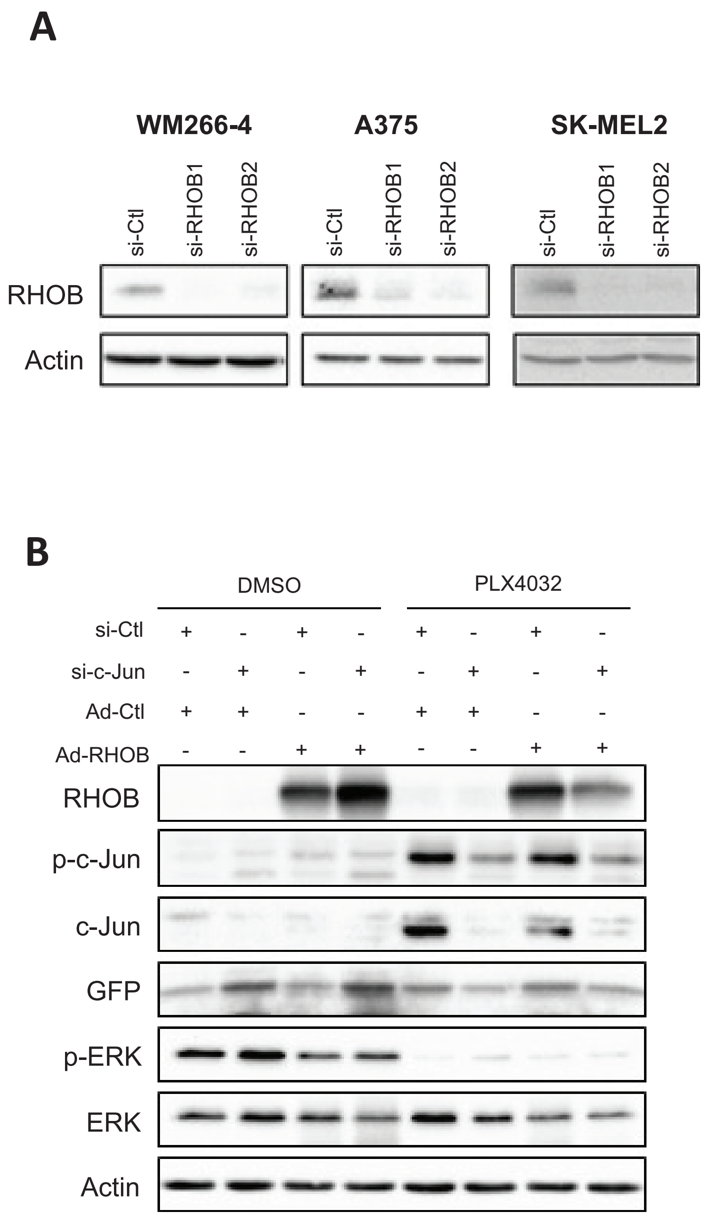

Supplementary Figure S3: Efficiency of RHOB and c-Jun silencing and of RHOB overexpression. A. Western blotting analysis of RHOB in WM266-4, A375 and SK-MEL2 cells transfected with RHOB-targeting (si-RHOB1 and si-RHOB2) or non-targeting (si-Ctl) siRNAs for 72 h. Actin was the loading control. **B.** Western blotting of the indicated proteins in WM266-4 transfected with c-Jun-targeting (si-c-Jun) or non-targeting (si-Ctl) siRNAs for 6 h and then transduced overnight with an adenovirus control (Ad-Ctl) or encoding RHOB (Ad-RHOB) before treatment with 1 µM PLX4032 for 72 h.

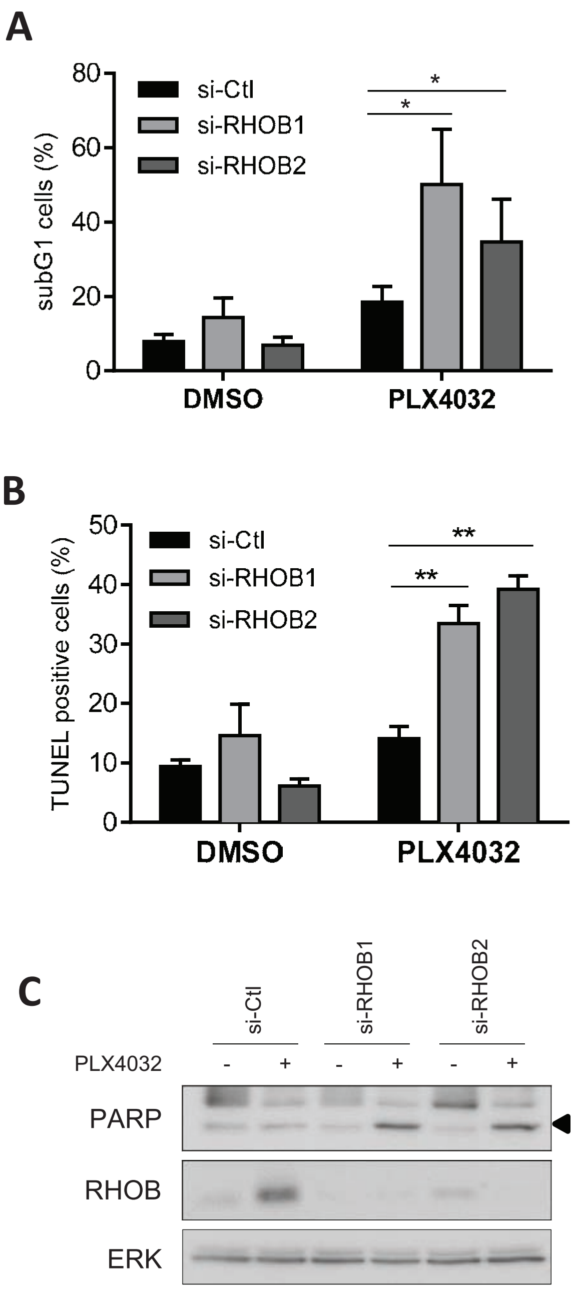

Supplementary Figure S4: Inhibition of RHOB sensitizes A375 cells to PLX4032-induced apoptosis. A375 cells were transfected with siRNAs control (si-Ctl) or targeting RHOB (si-RHOB1 and si-RHOB2) before treatment with 2 µM PLX4032 for 72 h. Cells were analyzed for cell cycle by FACS and subG1 was quantified **A.** or labeled for TUNEL assay **B.** or lysed for Western blotting **C.**

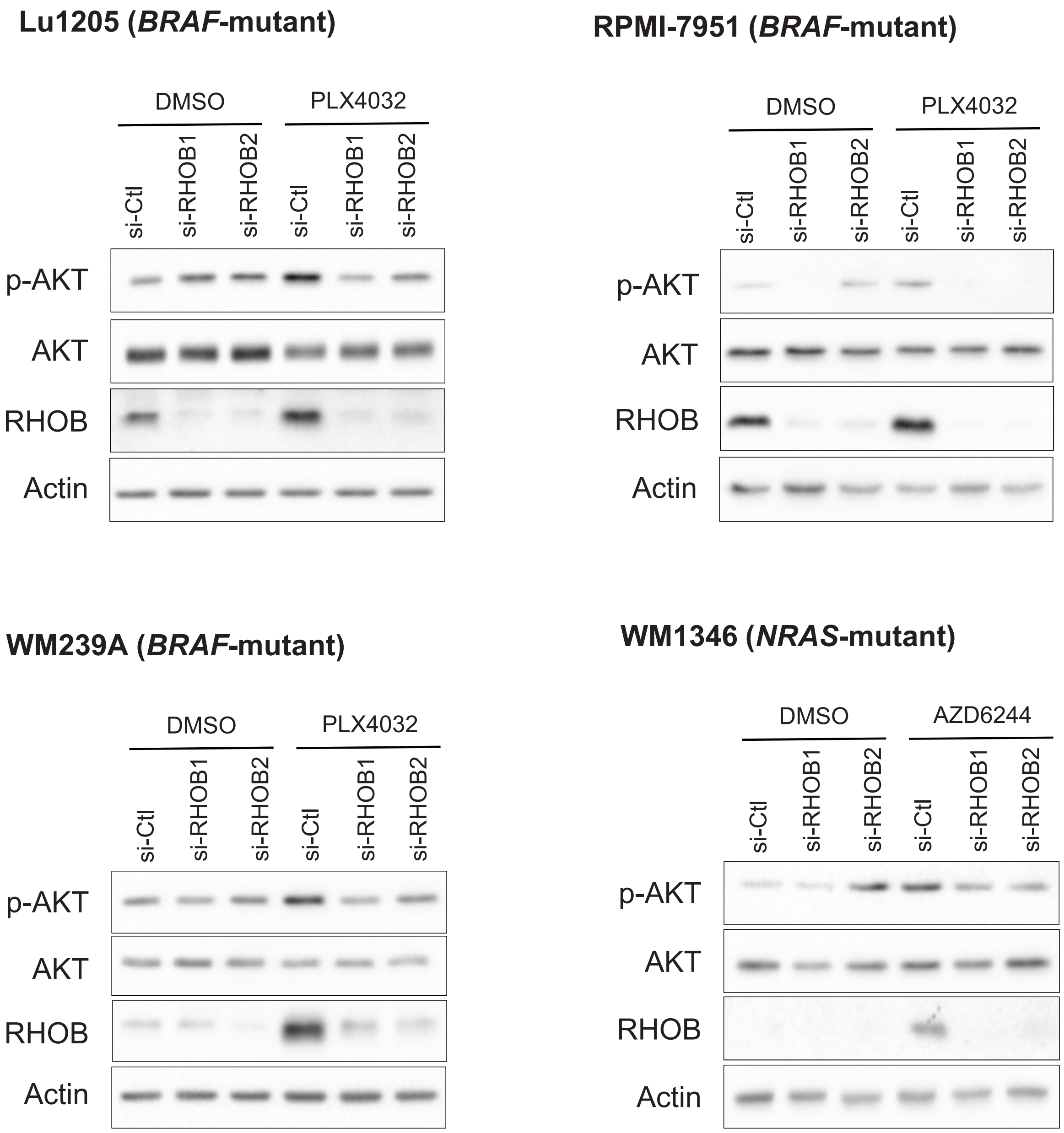

Supplementary Figure S5: Expression of phosphorylated AKT upon RHOB knockdown with siRNA during a MAPK inhibitors treatment. *BRAF*-mutant melanoma cell lines (Lu1205, RPMI-7951, WM239A) and *NRAS*-mutant melanoma cell lines (WM1346) were transfected with siRNA control (si-Ctl) or targeting RHOB (si-RHOB1 or si-RHOB2) before treatment with 1 µM PLX4032 (*BRAF*-mutant cells) or AZD6244 (*NRAS*-mutant cells) for 48 h. AKT phosphorylation was analyzed by Western blotting. Total AKT and RHOB were examined in parallel. Actin was the loading control.

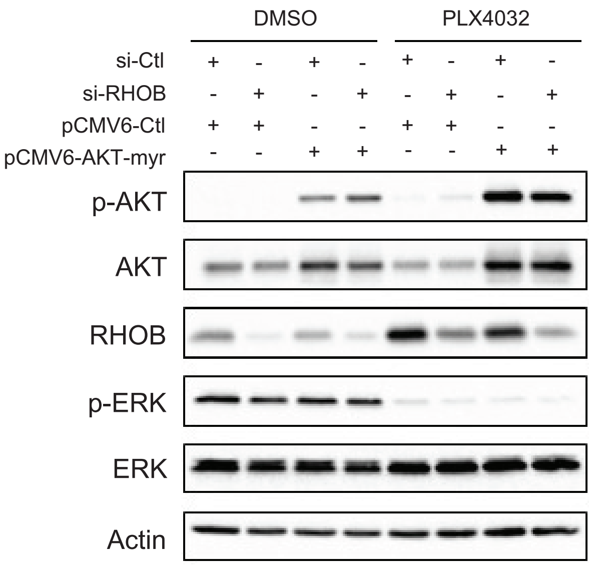

Supplementary Figure S6: Expression and activation of AKT upon RHOB siRNA and AKT-myr transfection. Western blotting analysis of the indicated proteins in WM266-4 cells co-transfected for 24 h with siRNAs control (si-Ctl) or targeting RHOB (si-RHOB2) together with an empty plasmid (pCMV6-Ctl) or a plasmid encoding AKT-myr (pCMV6-AKT-myr) before treatment with 2 µM PLX4032 for 72 h.

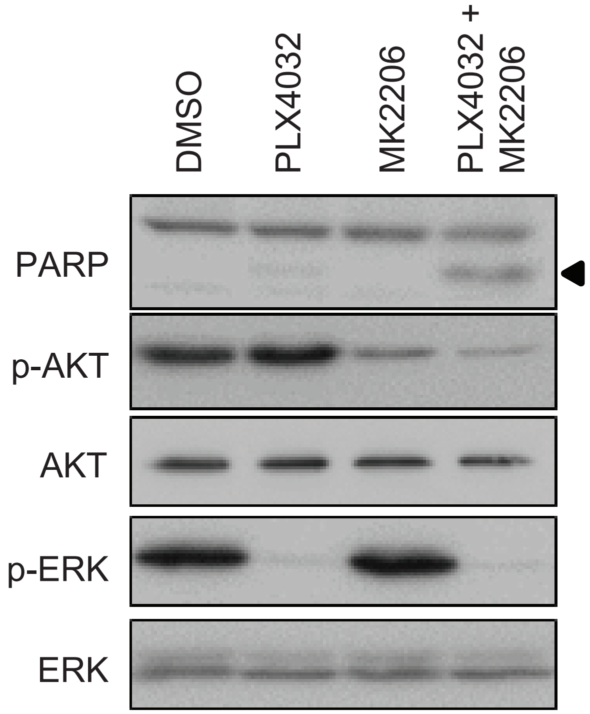

Supplementary Figure S7: PLX4032/MK2206 combination potentiates PARP cleavage in WM266-4 cells. Western blotting of indicated proteins in WM266-4 cells treated with 2 µM PLX4032 and/or 1 µM MK2206 (AKTi) for 48 h. The arrowhead at right indicates the cleaved fragment of PARP.

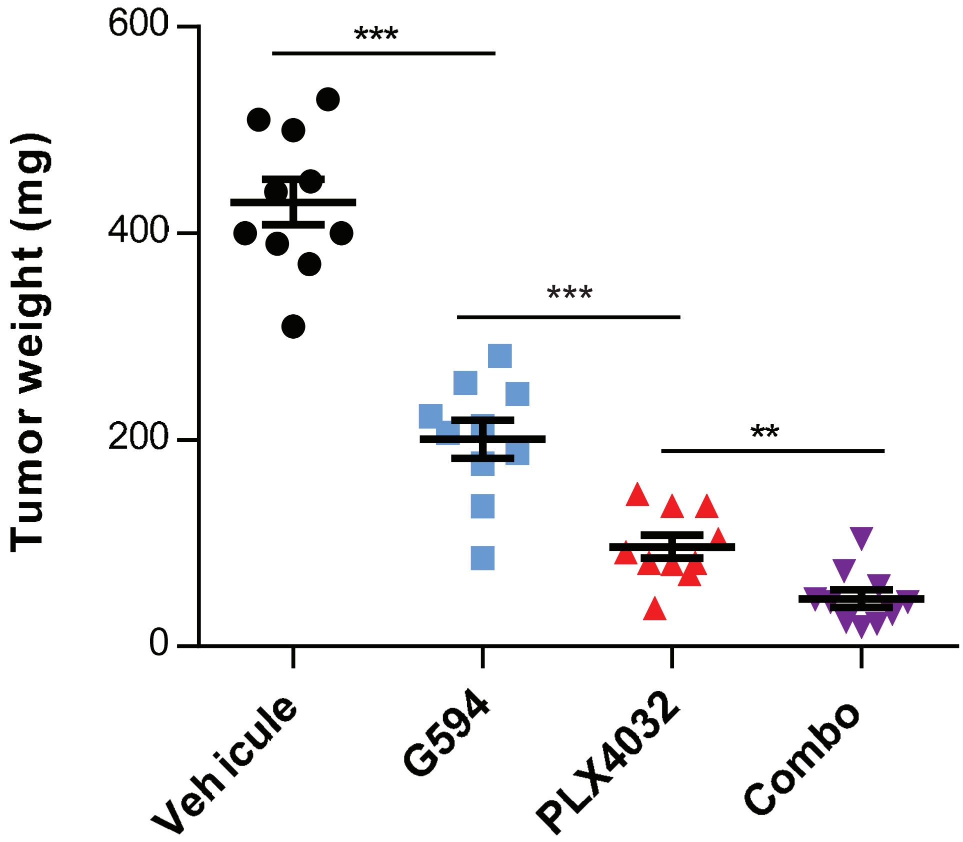

Supplementary Figure S8: Tumor weight analysis in mice treated with AKT and/or BRAF^{V600E} inhibitors. Athymic mice were subcutaneously inoculated with 1.5×10^6 WM266-4 cells and tumor allowed to grow for 10 days. Mice were randomized into groups (10 mice per group) and treated orally with vehicle, G594 or/and PLX4032 for 21 days. At the end of the experiment, tumors were harvested and weighted.

Supplementary Table S1. Dose-response curve analysis with GraphPad software

Cell line	Drug	siRNA	logIC50	IC50 (µM)	*p*-value compared to si-Ctl
WM266-4	PLX4032	Ctl	0.053 ± 0.063	1.13	
		RHOB1	– 0.471 ± 0.056	0.338	<0.0001
		RHOB2	– 0.551 ± 0.051	0.281	<0.0001
	AZD6244	Ctl	– 0.448 ± 0.053	0.357	
		RHOB1	– 0.842 ± 0.040	0.144	<0.0001
		RHOB2	– 0.710 ± 0.064	0.195	<0.005
A375	PLX4032	Ctl	0.475 ± 0.088	2.983	
		RHOB1	– 0.087 ± 0.064	0.819	<0.0001
		RHOB2	– 0.055 ± 0.053	0.277	<0.0001
	AZD6244	Ctl	– 0.609 ± 0.132	0.246	
		RHOB 1	– 1.083 ± 0.055	0.083	<0.005
		RHOB 2	– 0.945 ± 0.070	0.114	<0.005
SK-MEL2	AZD6244	Ctl	– 1.324 ± 0.044	0.047	
		RHOB1	– 1.886 ± 0.032	0.013	<0.0001
		RHOB2	– 1.855 ± 0.045	0.014	<0.0001

Supplementary Table S2. Dose-response curve analysis with GraphPad software

siRNA	logIC50	IC50 (μM)	*p*-value compared to si-Ctl
Ctl	0.827 ± 0.114	6.72	<0.0001
c-Jun	– 0.21 ± 0.031	0.610	

Supplementary Table S3. Dose-response curve analysis with GraphPad software

<table>
<tr><th>siRNA</th><th>Adenovirus</th><th>logIC50</th><th>IC50 (µM)</th><th>p-value compared to Ad-Ctl</th></tr>
<tr><td>Ctl</td><td>Ad-Ctl</td><td>− 0.401 ± 0.149</td><td>0.40</td><td rowspan="2">ns</td></tr>
<tr><td>Ctl</td><td>Ad-RHOB</td><td>− 0.218 ± 0.163</td><td>0.61</td></tr>
<tr><td>c-Jun</td><td>Ad-Ctl</td><td>− 0.553 ± 0.076</td><td>0.28</td><td rowspan="2"><0.001</td></tr>
<tr><td>c-Jun</td><td>Ad-RHOB</td><td>− 0.113 ± 0.087</td><td>0.77</td></tr>
</table>

Supplementary Table S4. Dose-response curve analysis with GraphPad software

siRNA	Plasmid	logIC50	IC50 (μM)	*p*-value compared to si-Ctl
Ctl	pCMV6-Ctl	0.174 ± 0.071	1.492	<0.0001
RHOB2	pCMV6-Ctl	– 0.468 ± 0.061	0.3407	
Ctl	pCMV6-Akt-myr	0.2818 ± 0.099	1.913	ns
RHOB2	pCMV6-Akt-myr	0.340 ± 0.120	2.189	

Supplementary Table S5. Patient's characteristics and RHOB staining analyzed by IHC

Patients (*n* = 31)	Total	RHOB pos. staining	RHOB neg. staining
Age (years ; median [IQR])	63.5 [42; 72]	72 [58; 77]	62 [40; 70]
Sex ratio (M/F)	1.46	0.6	2
AJCC 2009 staging IIIc, (*n*=)	8	1	7
IV M1a (*n* =)	0	0	0
M1b (*n* =)	19	5	12
M1c (*n* =)	7	2	5
Progression-Free Survival (days ; median [IQR])	217 [150 ; 285]	135 [105 ; 141]	235 [214 ; 314]

Pos. : positive; neg. : negative; M/F : male/female; IQR : interquartile range

Supplementary Table S6. Combination indexes and synergy analyzes on a panel of melanoma cell lines

Cells	Mutations	Akt inhibitor	MAPK inhibitors	Ci/doses		Synergy
				1 µM	3 µM	
**Lu1205**	BRAF	G-594	PLX4032	0.64	0.58	+
**SK-MEL28**	BRAF	G-594	PLX4032	0.37	0.5	+
**WM983B**	BRAF	G-594	PLX4032	0.65	0.48	+
**WM239A**	BRAF	G-594	PLX4032	0.21	0.16	++
**A375**	BRAF	G-594	PLX4032	0.38	0.55	+
**501Mel**	BRAF	G-594	PLX4032	0.34	0.35	+
**RPMI-7951**	BRAF	G-594	PLX4032	0.39	0.56	+
**WM1346**	NRAS	G-594	AZD6244	0.32	0.39	+
**SK-MEL2**	NRAS	G-594	AZD6244	0.31	0.44	+

Melanoma cell lines (2 *NRAS*-mutant or 7 *BRAF*-mutant) were treated with equal amount of AKT inhibitor (G594) and MAPK inhibitors (PLX4032 or AZD6244). Cell viability was measured 72 h later by MTS assay. Combination indexes (CI) were calculated with CompuSyn (strong synergism (++) 0.1 < CI < 0.3; synergism (+) 0.3 < CI < 0.7).

Supplementary Table S7. Sequences of the primers used for RT-qPCR

	Sequence / reference	Provider
si-Ctl	SR-CL000-005	Eurogentec
si-RHOB1	GGCAUUCUCUAAAGCUAUG-TT	Eurogentec
si-RHOB2	GCUAAGAUGGUGUUAUUUA-TT	Eurogentec
si-c-Jun	SMARTpool<br>1 : UGGAAACGACCUUCUAUGA<br>2 : UAACGCAGCAGUUGCAAAC<br>3 : GAGCGGACCUUAUGGCUAC<br>4 : AAGUCAUGAACCACGUUAA	Thermo Scientific

Supplementary Table S8. Sequences of the siRNA

Gene	Sequence	Reverse sequence
RHOA	TGGAAGATGGCATAACCTGTC	AACTGGTGGCTCCTCTGG
RHOB	TTGTGCCTGTCCTAGAAGTG	CAAGTGTGGTCAGAATGCTAC
RHOC	TGTCATCCTCATGTGCTTCTC	GTGCTCGTCTTGCCTCAG
RHOD	GATTGGAGCCTGTGACCTAC	GTAATCCGCCGCCAGAAG
RHOE/RND3	CCTGCTCCTCTCGCTCTC	TCTGGCTGGCTCTTCTCTC
RHOF	CAGACAGACCTCACGACAG	GAGTTCCAGAATGTTCCAAGAG
RHOG	CCGCTCTCACTTCCTTCTC	ACCACCACGCACTTGATG
RHOH	TTCACCTCCGAGACCTTCC	GCCACAGAGTAGCACATCAG
RHOJ	QuantiTect Primer Assay (Qiagen)	
RHOQ	TATGCCAACGACGCCTTC	GCCGTGTCATAGAGTCCTAG
RHOV	CATAGCAAGTAGTAGGCAGGAG	TCAGAGTGGGCAGTTAGAGG
RHOU	CGGTGGTGTCTGTGGATG	GAAGATGTCTGTGTTGGTGTAG
RND1	GCAAGTGTTAGCGAAGGATTG	GCAGAGTGGACGGACATTATC
RHON/RND2	QuantiTect Primer Assay (Qiagen)	
CDC42	GTCAAGTATGTGGAGTGTTCTG	CACCTGCGGCTCTTCTTC
RAC1	AGAACACCGAGCACTGAAC	ACGCATCTGAGAACTACATAGG<br>CCTATGTAGTTCTCAGATGCGT
RAC1b	TACGGTAAGGATATAACCTCCC	ACCTCAGGATACCATCTTTGC<br>GCAAAGATGGTATCCTGAGGT
RAC2	GGACAGCAAGCCAGTGAAC	GGAGAAGCAGATGAGGAAGAC
RAC3	GTGATGGTGGACGGGAAAC	CACTTGGCACGAACATTCTC
Actin	TCCCTGGAGAAGAGCTACGA	AGGAAGGAAGGCTGGAAGAG
